# Preparation and Characterization of Li-Ion Graphite Anodes Using Synchrotron Tomography

**DOI:** 10.3390/ma7064455

**Published:** 2014-06-12

**Authors:** Tim Mitsch, Yvonne Krämer, Julian Feinauer, Gerd Gaiselmann, Henning Markötter, Ingo Manke, Andreas Hintennach, Volker Schmidt

**Affiliations:** 1 Deutsche ACCUmotive GmbH & Co. KG, Neue Straße 95, Kirchheim unter Teck 73230, Germany; E-Mails: yvonne.kraemer@daimler.com (Y.K.); julian.feinauer@daimler.com (J.F.); 2 Institute of Stochastics, Ulm University, Helmholtzstr. 18, Ulm 89069, Germany; E-Mails: gerd.gaiselmann@uni-ulm.de (G.G.); volker.schmidt@uni-ulm.de (V.S.); 3 Helmholtz-Zentrum Berlin, Hahn-Meitner-Platz 1, Berlin 14109, Germany; E-Mails: henning.markoetter@helmholtz-berlin.de (H.M.); manke@helmholtz-berlin.de (I.M.); 4 Daimler AG, HPC H152, Mercedesstr. 137, Stuttgart 70367, Germany; E-Mail: andreas.hintennach@daimler.com (A.H.)

**Keywords:** graphite, synchrotron tomography, Li-ion, preparation, degradation, porosity, tortuosity, multi-layer

## Abstract

We present an approach for multi-layer preparation to perform microstructure analysis of a Li-ion cell anode active material using synchrotron tomography. All necessary steps, from the disassembly of differently-housed cells (pouch and cylindrical), via selection of interesting layer regions, to the separation of the graphite-compound and current collector, are described in detail. The proposed stacking method improves the efficiency of synchrotron tomography by measuring up to ten layers in parallel, without the loss of image resolution nor quality, resulting in a maximization of acquired data. Additionally, we perform an analysis of the obtained 3D volumes by calculating microstructural characteristics, like porosity, tortuosity and specific surface area. Due to a large amount of measurable layers within one stacked sample, differences between aged and pristine material (e.g., significant differences in tortuosity and specific surface area, while porosity remains constant), as well as the homogeneity of the material within one cell could be recognized.

## 1. Introduction

In recent times, electrochemical energy storage has become more important, especially for usage in e-mobility applications, such as pure electrical, plug-in-hybrid or mild-hybrid vehicles. The requirements regarding long-life (usage time ≥ 10 years) and high energy density are dominantly fulfilled by Li-ion cells. Furthermore, due to their complicated aging behavior [1,2], they are the focus of many researchers for gaining better understanding of the aging process.

During the lifetime of Li-ion cells, a lot of aging mechanisms [1,2] occur, which affect different components, like the anode or cathode active material, separator, current-collector or electrolyte. These mechanisms interact in a very complex way. Notably, graphite, which is mainly used as an anode material, is involved in many aging processes [1]. The literature dominantly shows well-known, but only partly understood mechanisms, like the growth of the SEI (solid electrolyte interface) at the boundary between graphite particles and the electrolyte [3–5], lithium deposition caused by high current or low temperature charging [6–8], micro-cracking of graphite-particles caused by massive electrical usage [9] and structural changes of the anode active material due to multiple reasons.

To get a closer insight into the microstructure of the anode active material, we use synchrotron tomography [10–19]. Besides the qualitative impressions that one can get from the visual inspection of tomographic 3D images, we calculate the structural characteristics of the samples to obtain a quantitative statement of the state of the material samples. In particular, we look at the spherical contact distribution function for the graphite material, which is closely related to the diffusive behavior of the graphite phase. Furthermore, we calculate the tortuosity, which is an important characteristic related to the transport of ions in porous media [20]. A change in the tortuosity for degraded material can be explained by cracks and fractures in the structure.

The paper is organized as follows. In Section 2, we give an overview of the necessary steps for the disassembling of different types of Li-ion cells. Furthermore, a promising approach for the surface modification of graphite to qualify the presence of lithium deposition is introduced in Section 2.4. Accordingly, we discuss the preparation of samples to stacks to improve the efficiency of synchrotron tomography in Section 3. Up to ten samples can be stacked together, in order to be measured in parallel. The experimental setup, *i.e.*, the samples that are extracted from pristine and aged cells, as well as the necessary post-processing methods, like reconstruction and binarization, are presented in Section 4. Finally, in Section 5, we discuss the results of several structural analyses, like the calculation of tortuosity and spherical contact distribution functions. These analysis methods allow a quantitative description of the samples and show the potential of synchrotron tomography in combination with refined preparation techniques as a valuable tool for the investigation and characterization of functional materials.

## 2. Extraction of Samples

In this section, we describe the procedure to obtain anode samples from different types of Li-ion cells for structural analysis. For the purposes of comparability, all analyzed cells were discharged to 0% SOC (state-of-charge) using the CC-CV (constant current constant voltage) discharge procedure with the cut-off voltage given in the datasheet delivered by the manufacturer. The method for the extraction of samples from the anode material will be described in detail.

### 2.1. Cell Disassembly

To minimize degradation caused by the presence of oxygen and humidity, we disassembled all cells in a glovebox (mBraun MB-200B, H_2_O < 4 ppm, O_2_ < 4 ppm).

Automotive Li-ion pouch cells were opened with a ceramic scalpel to avoid unwanted shorts and further structural changes. After removal of the upper pouch foil, the electrodes can be separated and analyzed optically.

The aluminum housing of cylindrical cells was sliced next to the positive terminal using a self-constructed tool. Then, the positive terminal and its connection to the cathode active material were separately disassembled. Finally, the aluminum case has to be rolled down using small pliers. To ensure non-destructive disassembly, we controlled the temperature of the cell as the best indicator for shorts. The used setup consists of a PT100 thermal-resistor connected to a PicoTechology^®^ PT-104 data logger visualized by a common notebook. If temperature exceeded 35 °C, we did not use the cell for further analysis.

### 2.2. Sample Selection

**Figure 1.**
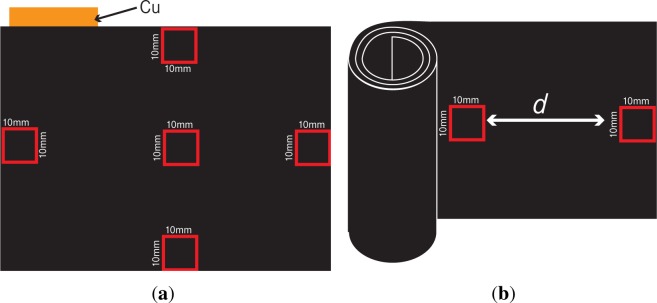
Anode sample selection from different types of cells. (**a**) Pouch cell; (**b**) cylindrical cell.

As shown in Figure 1(a), from one pouch cell, we extracted multiple samples with a size of 10 mm × 10 mm using a ceramic scalpel. In the case of cylindrical cells, several equally-sized (10 mm × 10 mm) samples were sliced from equidistant intervals *d* of the jellyroll; see Figure 1(b).

Subsequently, all samples were washed with dimethyl carbonate (DMC).

### 2.3. Separation of Graphite Layers

**Figure 2.**
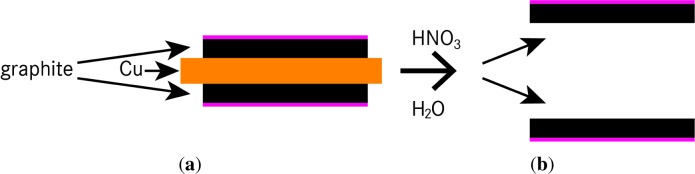
Separation of two graphite layers out of one sliced sample. (**a**) The structure of the anode layer; (**b**) the separated active material after the application of HNO_3_; 65%.

The structure of an anode layer used in Li-ion cells usually consists of a copper foil coated with a mixture of graphite and a binder on both sides; see Figure 2(a). Metals like copper have a high density, and therefore, X-ray beams used in synchrotron tomography are not able to pass through. To sustain better image quality from the anode sample, it is mandatory to separate the copper foil and graphite layers. Three different methods were compared. An overview is shown in Table 1.

### 2.4. Surface Modification

To achieve reproducible results, defined sample sizes and flat shapes are essential. Therefore, chemical treatment using nitric acid (HNO_3_; 65%) yielded the best graphite monolayer [10,21]. Depending on the thickness of the monolayers, the type of degradation (e.g., lithium deposition) and the binder used by the manufacturer, we received the best samples using 5 mL of demineralized water and three to ten drops of HNO_3_, resulting in a dilute nitric acid (2%–6%) solution. After 5–30 s, the copper foil dissolved. Both graphite layers were washed twice with demineralized water and once using propane-2-ol (C_3_H_7_OH), while constantly paying attention to the orientation of the layers (see the pink markers in Figure 2(b)). Finally, the separated layers were stored on a small sheet of paper for at least 10 min in order to dry.

Metallic lithium, which was formed as a result of electrochemical plating during the cycling of an electrode, is not visible in neutron-diffraction experiments. However, there are some paths to enhance the visibility by adding complexes and/or different metal-ions on the metallic lithium parts. With a surface modification using, e.g., glucosamines, the metallic lithium deposition can be made visible in neutron experiments.

The deposition of metallic lithium is primarily a diffusion-triggered process. To verify the proposed surface modification procedure using glucosamines, the cells were cycled 20 times (the 1 C charge and 1 C discharge current at a potential range of 3 V – 4.2 V) at an ambient temperature of −10 °C, to ensure the presence of metallic lithium on the anode surface.

The additives were used with a slight excess to ensure a homogeneous coating of the lithium-plated parts of the electrode. A homogeneous coating proved to be essential for the detailed investigation of the surface.

**Figure 3.**
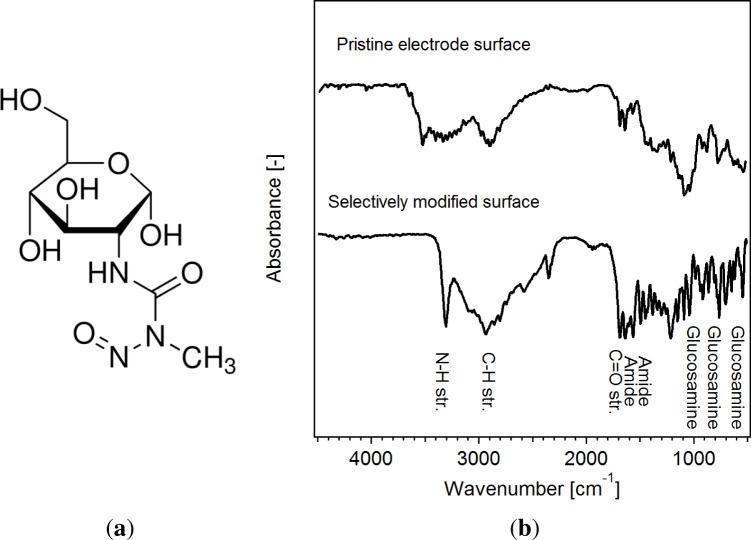
(**a**) *N*-(Methylnitrosocarbamoyl)-*α*-D-glucosamine; (b) FTIR spectra of a pristine electrode and the surface modified parts, which exhibited metallic lithium plating.

**Figure 4.**
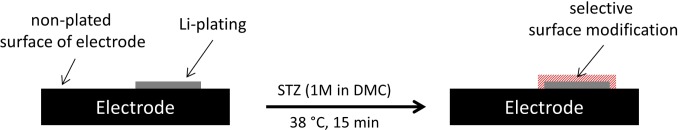
Schematic surface modification process.

*N*-(Methylnitrosocarbamoyl)-*α*-D-glucosamine (STZ; Sigma-Aldrich; see Figure 3(a)) was used for the selective modification of the surfaces *ex situ*. A solution of STZ in DMC (1M) was prepared in an argon-filled glovebox. About 2 wt% of a solution of predispersed surfactants (Triton X-109, Triton X-209; 1:1 by volume) in EC:DMC (1:1 by volume, 10 wt%) were added with stirring. This solution could be directly added into the electrolyte between the electrodes. For a homogeneous mixing of the additive with the electrolyte and to ensure a homogeneous wetting of the electrodes, it is important to allow a standing time of about 30 min after the injection of the STZ solution was completed. An adjacent heating step (38 °C, 15 min) initiated the surface modification. This process is schematically shown in Figure 4. Note that no electrochemical cycling was performed after the additive was added. This is the reason for the low electrochemical stability of the glucosamine, while the stability at open circuit potential is high enough for a safe preparation of the samples.

FTIR microscopy was applied for the investigation of the influence of the surface modification at lateral resolution, where an HJY LabRAM HR with an FTIR module was used.

While the upper spectrum of Figure 3(b) displays typical bands of the as-prepared electrode, including parts with metallic lithium, the lower spectrum in Figure 3(b) exhibits carbonyl peaks (C = O) at 1628 cm^−1^ and hydroxyl peaks (C-H) at about 3304 cm^−1^. Significant differences could be observed between parts of the electrode where lithium-plating and pristine parts occurred. With an adjacent mapping technique, larger areas of electrodes (about 1.5 cm × 1.2 cm) were investigated to validate the surface modifying effect of STZ. The LC-MS analysis of the electrolyte showed that the consumption of consumed STZ could be correlated very well with the amount of metallic lithium that was deposited onto the surface of the electrodes.

## 3. Multilayer Preparation

Synchrotron tomography is a useful tool to obtain the microstructural characteristics of the Li-ion anode material. To maximize the efficiency, we prepared the anode samples in a multilayer stack. This gives us the opportunity to compare different kinds of aged cells and various anode materials from different manufactures and to verify the homogeneity of the production processes.

Therefore, our approach is to stack the anode layers to measure several samples in parallel. This means that we obtain one image for all samples inside one stack. Hence, the anode samples inside one stack have to be divided sharply with a separator layer in between. The additional layers have to feature a non-particle-based microstructure for good visibility and contrast against graphite. In this work, we investigate the influence of different separation materials and stacking properties.

### 3.1. Separation Materials

Focusing on the microstructure, the following materials were selected:

1. adhesive tape (lattice structure of backing film);
2. Li-ion separator materials (microporous polymer membrane [22]);
3. cellulose papers (fabric structure).

Beside the discussed microstructural properties above, we identified the following characteristics, which are important for a promising stacking preparation: (1) thickness; (2) stability of the stack; (3) stickiness; and (4) sliceability.

The properties of the investigated materials are summarized in Table 2.

### 3.2. Layer Stacking

To realize a good resolution, the optimal sample dimensions for synchrotron tomography should be a cylinder (∅ 1 mm, h: 1 mm). Thus, the thickness of the complete stack can be calculated using the following formula:

$$d_{\text{Stack}}=n\cdot d_{\text{anode}-\text{layer}}+(n+1)\cdot d_{\text{separation}}$$

with *n* number of anode samples per stack, *d*_anode−layer_ the thickness of the anode layer and *d*_separation_ the thickness of the separation material.

Generally, stacking was performed by alternating separation layers (25 mm × 25 mm) and anode samples (10 mm × 10 mm). At the bottom and the top of the stack, a separation layer is essential to ensure stability. A maximum overlap occurs between all anode samples inside one stack. Furthermore, it is important to ensure the correct orientation (see Figure 2(b), pink marker) of each layer.

Note that for stack preparation using Li-ion separator materials and cellulose papers, additional rapid glue (LocTite^®^ 4850) based on cyanoacrylates was used. Each stack was marked on the top and stored for 24 h.

We assume that there is no effect on electrode morphology using cyanoacrylate-based glue. This was confirmed by comparison of adhesive tape and glue-based preparation methods; no significant differences could be noticed.

### 3.3. Stack Slicing and Final Setup

**Figure 5.**
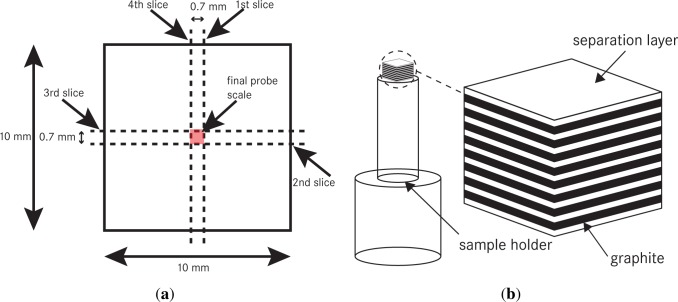
(**a**) The necessary cuts of the stacked probe to realize the final geometrics; (**b**) the final preparation setup.

As described above, the final geometry of the anode stack should be cylindrical. To achieve an approximation of 1 mm in diameter, we applied a rectangular shape. By using the Pythagorean theorem, the length of the edge was calculated to be 0.7 mm. The sequence of the slices is shown in Figure 5(a).

**Figure 6.**
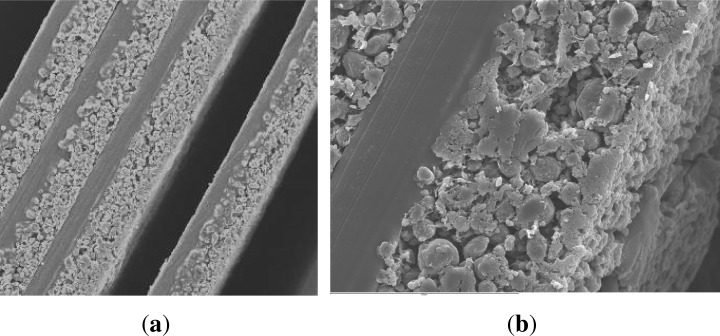
Through-plane SEM images of a prepared anode stack. (**a**) Cutout with four layers; (**b**) cutout of two layers and the separator.

Afterwards, we were able to monitor the size of the stack by using an optical microscope (Leica) or SEM imaging; see Figure 6. Finally, the prepared anode stack was fixed on a specific sample holder with a little amount of hot or rapid glue to perform synchrotron measurement. Figure 5(b) shows the final probe, which was applied to the tomography setup.

### 3.4. Discussion

The experimental results showed significant differences among the investigated separation material groups; see Table 2.

Double-sided adhesive tape showed a thickness of 35 μm, and the maximum number of the anode layer was obtained. The stability was very high, but due to a missing carrier, a stack made from this material could not be sliced. Single-sided adhesive tape exhibits the opposite behavior.

Stacks consisting of microporous polymer membranes led to the highest number of anode layers. However, an additional primer (LocTite^®^ 770) was required, because of the poor adhesive properties of polypropylene (PP) and polyethylene (PE). Despite the application of the primer, the stability of the anode layer stack was not sufficient.

The three investigated cellulose papers showed very good stacking and slicing characteristics, only differing in thickness and, therefore, in the amount of maximum anode layers per stack. As the best compromise between the maximum number of layers and stability, we selected greaseproof paper as the separation material for all further stack preparations.

## 4. Experimental Section

### 4.1. Synchrotron Tomography

**Figure 7.**
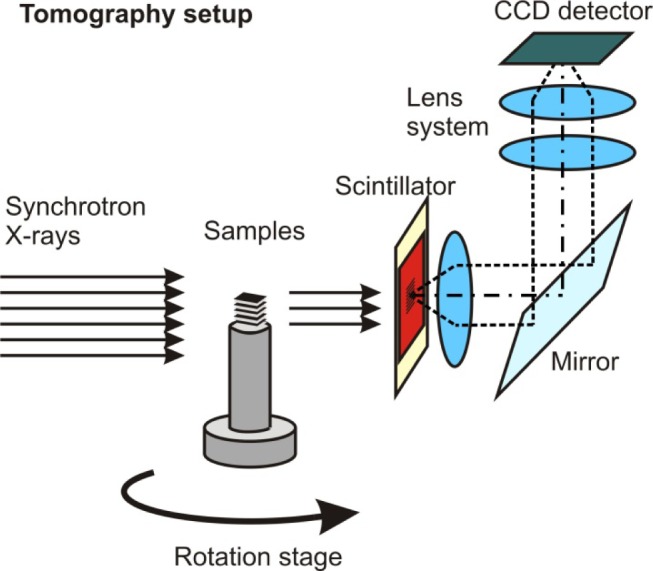
Setup used for synchrotron tomography.

The synchrotron X-ray tomography measurements were performed at the imaging station of the BAMline [23,24]. The facility is located at the electron storage ring, BESSY II, at Helmholtz Centre, Berlin. A monochromatic synchrotron beam at an energy level of 19 keV was obtained by a W-Si multilayer monochromator with an energy resolution of about ΔEE=10−2. The X-ray energy was adapted to the thickness and absorption properties of the investigated samples. It was found that 19 keV is a good compromise between transmission intensity and contrast. A CWO scintillator with a thickness of 50 μm was used to convert the X-rays into visible light. A PCO camera with a 4008 × 2672 pixel CCD chip was employed to capture the images. An optical setup (“Optique-Peter”) was used to transfer the light onto the CCD chip of the camera system [25]; see Figure 7.

The used pixel size was 0.44 μm and the achieved spatial resolution about 1 μm. The field of view was about 1.7 × 1.2 mm^2^.

**Figure 8.**
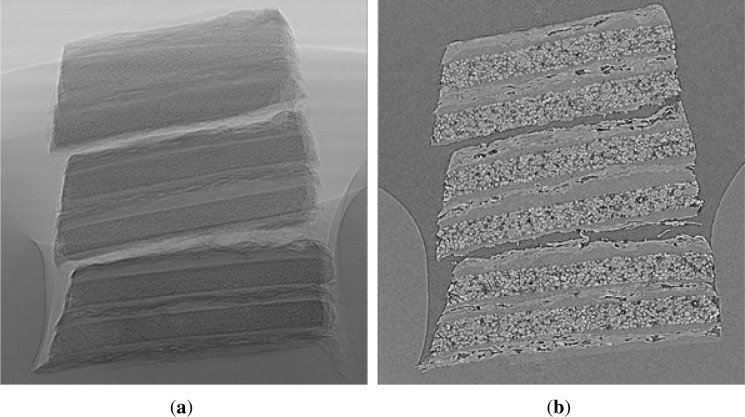
2D images of a sample stack. (**a**) Radiographic projection image; (**b**) reconstructed image.

A set of 2200 radiographic images were taken from the samples over an angular range of 180°. Additionally, 230 flat field images (*i.e.*, without a sample) were taken. After subtraction of the dark field signal, the radiographic projections were divided by the flat field images in order to obtain bright field-corrected (normalized) images (see Figure 8(a)). The exposure time for each radiographic projection was 3 s. The time for a complete tomographic measurement was about three hours.

A proper normalization provides the transmission of X-rays through the sample according to the Beer–Lambert law:

$$\frac{I}{I_{0}}=e^{\sum \mu \cdot d}$$

Here, *I*_0_ and *I* denote the intensity of the beam in front of and behind the sample, *d* the transmitted distance through a certain material and *μ* the linear attenuation coefficient of that material at the used X-ray energy.

### 4.2. Data Post-Processing

The information of the transmission was used for the three-dimensional reconstruction of the attenuation coefficients of each voxel in the sample volume. This was done with a standard algorithm, the filtered-back projection [26]. Therefore, the images were projected back into the volume according to the projection angle. This was applied for all angular steps. As a result, the object would have been blurry. To avoid this, a high-pass filter was applied to each projection in the horizontal frequency domain (Hamming filter) before back-projection. A vertical slice through the reconstructed volume is shown in Figure 8(b).

**Figure 9.**
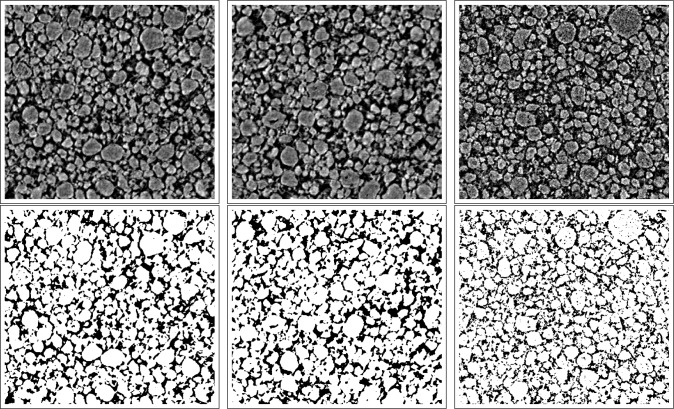
2D slices from the reconstructed grayscale (first row) and the binary (second row) images of $P_{C}^{1}$ (**left**), $P_{E}^{2}$ (**center**) and $D_{C}^{1}$ (**right**).

Since the contrast in the 3D synchrotron images is very high, we binarized those by global thresholding [27,28]. The 8-bit grayscale threshold is chosen to 32 for pristine and 72 for the degraded electrodes in order to obtain reasonable porosities between [0.22, 0.26] for the samples. Figure 9 shows the effect of binarization.

## 5. Structural Analysis

In this section, we compute several structural characteristics for images of Li-ion cells obtained by the preparation and visualization methods discussed in Sections 2–4. This enables us to perform a quantitative comparison and a discussion of different electrode samples. Note that the considered characteristics are known to be linked to the functionality of graphite electrodes. The analysis addresses two main questions that play an important role in the investigation of Li-ion cells:

1. Can the microstructure of graphite be regarded as statistically homogeneous over the whole cell?
2. Can the influence of aging on the microstructure of graphite be characterized?

To answer these questions, we take three scenarios into account where two synchrotron images for each scenario are considered. In particular, the scenarios are:

(i) pristine material from the center of the cell;
(ii) pristine material from the edge of the cell;
(iii) degraded material from the center of the cell.

In the following, we denote the binary images considered for Scenarios (i)–(iii) by $P_{C}^{1},P_{C}^{2},P_{E}^{1},P_{E}^{2},D_{C}^{1}\;\text{and}\;D_{C}^{2}$, where P (D) stands for pristine (degraded) electrodes and C (E) for cutouts at center (edge) regions; *cf*. Section 2 and Figure 1(a). Recall that these images are gained as described in Sections 2–4. For a sample of each of the three groups, see Figure 9. Note that the considered cutouts have approximately the same dimension of 660 × 550 × 50 μm^3^.

**Figure 10.**
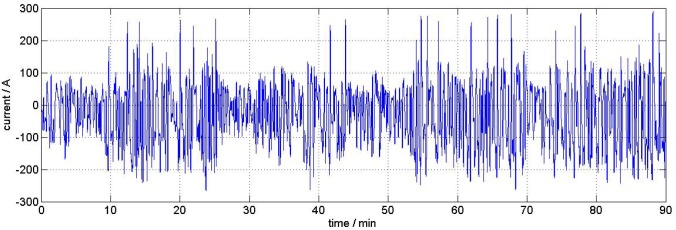
Current profile applied to the cyclically-degraded cell.

The samples analyzed in this section are extracted out of a big-sized automotive EVpouch cell (50 Ah) nominal capacity, NMC-cathode, potential range 3 V – 4.2 V). The degraded cell was heavily cycled for about nine months with a time-scaled realistic load profile (see Figure 10) similar to usage in a purely electric vehicle, at an ambient temperature of 25 °C. The final cell capacity was 70% of the initial capacity, measured with a 1 C-discharge-current $\left(1C=\frac{{\text{Capacity}}_{\text{nom}}}{1h}\right)$.

The detailed structural analysis explained below was possible due to the preparation and visualization techniques proposed in this paper.

### 5.1. Comparison of Structural Characteristics

The goal of this section is to obtain a quantitative comparison of the binary images, $P_{C}^{1},P_{C}^{2},P_{E}^{1},P_{E}^{2},D_{C}^{1}\;\text{and}\;D_{C}^{2}$, by computing several structural characteristics for each of these images.

As a first structural characteristic, we consider the porosity, which is the fraction of the volume of voids (*i.e.*, the volume of pore space) over the total volume [29]. The second characteristic is the specific surface area, which specifies the total surface area of a material per unit volume [29].

The results obtained for the porosity and the specific surface area are listed in Table 3. It turns out that the porosities of all considered samples are nearly identical. The same holds for the specific surface areas of the pristine electrodes $\left(P_{C}^{1},P_{C}^{2},P_{E}^{1},P_{E}^{2}\right)$, whereas, contrarily, the specific surface areas of degraded electrodes are significantly higher than their counterparts of pristine electrodes. The microstructural characteristics of both degraded samples $\left(D_{C}^{1},D_{C}^{2}\right)$ exhibit an almost perfect match.

For a more detailed characterization of the graphite and pore space, respectively, we consider the probability density function of so-called spherical contact distances from the pore to the graphite phase and *vice versa* [29]. This characteristic can be interpreted as some kind of pore (particle) size distribution. The spherical contact distance of a point located in the pore phase or the graphite phase, respectively, is given by the minimum distance to the complementary phase. Note that the considered density functions uniquely determine the probability that the spherical contact distance of a randomly chosen point located in the pore phase or the graphite phase, respectively, is within a certain interval. In summary, the spherical contact distance distribution provides a good measure for the ’typical’ distances from the pore to graphite phase and *vice versa*; *cf*. [29].

The computed probability density functions for the spherical contact distances from the graphite to pore phase and *vice versa* are visualized in Figure 12(a) and 12(b), respectively. The corresponding mean values and variances are listed in Table 4. It turns out that the density functions for the spherical contact distances computed for $\left(P_{C}^{1},P_{C}^{2},P_{E}^{1},P_{E}^{2}\right)$ nearly coincide, whereas a large discrepancy is observed between the results for pristine and degraded electrodes. In particular, for both the spherical contact distances from the graphite to pore phase and *vice versa*, these distances are by trend smaller for the degraded electrodes compared to the pristine electrodes. This coherence can be explained by cracks and fractures in the microstructures of degraded electrodes. These deformations lead to a much finer dispersed graphite phase within the degraded electrodes, whereas the graphite phase in the pristine electrodes is much more aggregated. This assumption is also supported by the visual impression of Figure 9.

**Figure 11.**
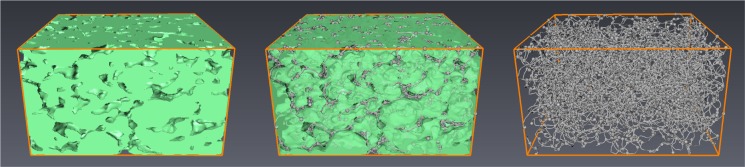
Extraction of the pore space graph via skeletonization from a 3D binary image: binary image (**left**); solid phase and pore space graph (**center**); pore space graph (**right**).

Finally, we focus on the so-called geometric tortuosity; see e.g., [30–32]. It evaluates the tortuosity of the pathways through the pore phase in the through-plane direction. In particular, starting from a randomly chosen location on top of the porous material, the geometric tortuosity is defined as the random Euclidean length of its shortest path through the material along all possible paths within the pore space divided by the material thickness (in the *z*-direction). For this purpose, the set of pore space paths is represented by a geometric 3D graph. This graph is computed using the skeletonization algorithm implemented in the software, Avizo 7; see Figure 11.

**Figure 12.**
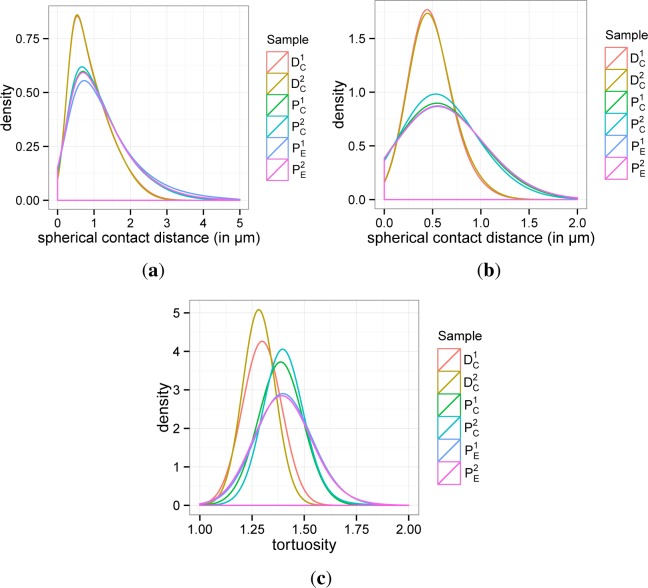
Probability density functions for the structural characteristics of graphite layers. (**a**) Spherical contact distances from the graphite to pore phase; (**b**) spherical contact distances from the pore to graphite phase; (**c**) geometric tortuosity.

The computed probability density functions for the geometric tortuosity are visualized in Figure 12(c), whereas the corresponding mean values and variances are listed in Table 4. As a result, we again obtain that the differences of geometric tortuosity within both groups (*i.e.*, pristine $P_{C}^{1},P_{C}^{2},P_{E}^{1},P_{E}^{2}$ and degraded $D_{C}^{1},D_{C}^{2}$ electrodes) are negligible. Moreover, there exist significant deviations between the two groups, where the degraded electrodes have significantly smaller values of the length of the shortest pathways through their pore space. This can be again explained by the much finer, dispersed graphite phase within degraded electrodes.

### 5.2. Summary

In this section, we summarize the discussion of the results obtained by the structural analysis. It turns out that for all considered characteristics, the differences within the pristine and degraded groups are negligible, whereas a significant discrepancy between the two groups can be observed. In particular, we can conclude that it does not matter from which region of the tomograms the cutouts are extracted. This also indicates that the considered materials are perfectly homogeneous. Moreover, because of the structural differences between pristine and degraded electrodes, we can conclude that synchrotron tomography is an adequate method for detecting such changes. Thus, the proposed preparation and visualization techniques described in Sections 3 and 4 provide an excellent tool for a cost- and time-saving analysis of degradation processes in the microstructure of Li-ion cells.

## 6. Conclusions

We successfully introduced a new preparation method for the analysis of Li-ion graphite material using synchrotron tomography. The complete procedure, including cell disassembly, sample selection and extraction, as well as the proposed efficient multilayer stacking, were described in detail.

Due to the discussed complex aging behavior of Li-ion cells, many investigations have to be done to gain a more detailed understanding. Particularly, the anode material is a key factor for cell-performance and the limitation of the lifetime. Since the microstructure of the active material is essential for aging characteristics, synchrotron tomography is an excellent method, because the resolution is high enough to detect the shape of particles and the differences between particles and pores in all three dimensions.

The presented preparation method extends the advantages of synchrotron tomography by massively parallel measurement of samples. This results in the possibility of comparing different regions of a given cell, enhancing statistical data due to analyzing many samples from a similar area of a cell, comparing anode material from different manufactures or cells and in the lowering of costs, because less measurements are necessary.

As shown in Section 5, structural analysis pointed out that aging causes significant changes in the microstructure of graphite material. Furthermore, we found out that the investigated samples from the same cell do not significantly differ from a statistical point of view. Hence, the method provides the possibility to analyze the homogeneity of the used active material. On the other hand, the difference between pristine and aged cells, with respect to the calculated characteristics, e.g., the tortuosity and sizes of pores and particles, is significant, which leads us to more detailed analyses and investigations, like (functional) particle-based modeling, to be done as future work. Furthermore, the structural characteristics of lithium deposition, which can be made visible in synchrotron tomography using the method proposed in Section 2.4, will be investigated in a forthcoming paper.

## Figures and Tables

**Table 1.** Methods for the separation of the graphite from the copper foil.

Tested methods	Sample size	Shape
Scrape off Cu	Undefined	crimped
Freeze with N_2_ and scrape off Cu-metal	Undefined	crimped
Chemical treatment with (HNO_3_; 65%)	defined	flat

**Table 2.** Overview of the separation materials and stack characteristics.

Material	Separator thickness	Max. layers [Table-fn tfn1-materials-07-04455]	Stability of stack	Stickiness		Sliceability
				Add. Glue	Property	
Single-sided adhesive tape	45 μm	9	−	no	no	++
Double-sided adhesive tape	35 μm	10	++	no	no	− −
Celgard(r) 2325	25 μm	11	− −	yes	primer	0
Celgard(r) 2400	25 μm	11	− −	yes	primer	0
Celgard(r) 2500	25 μm	11	− −	yes	primer	0
Greaseproof paper	60 μm	7	++	yes	good	+
Wrapping tissue	35 μm	10	+	yes	good	+
Reprographic paper	100 μm	5	++	yes	good	+

assuming an anode-thickness of 60 μm.

**Table 3.** Porosity and specific surface area computed for six selected binary images of anode layers.

Characteristic	Sample					
	$P_{C}^{1}$	$P_{C}^{2}$	$P_{E}^{1}$	$P_{E}^{2}$	$D_{C}^{1}$	$D_{C}^{2}$
Porosity	0.267	0.268	0.257	0.272	0.244	0.259
Specific surface area (1/μ*m*)	0.435	0.44	0.416	0.434	0.594	0.588

**Table 4.** Mean and variance of the spherical contact distances for the pore (scdf_P_) and graphite phase (scdf_graphite_) in μm, as well as of the geometric tortuosity (tort) computed for six selected binary images of anode layers.

Characteristic	Sample					
	$P_{C}^{1}$	$P_{C}^{2}$	$P_{E}^{1}$	$P_{E}^{2}$	$D_{C}^{1}$	$D_{C}^{2}$
Scdf_P_ mean	1.176	1.152	1.275	1.184	0.92	0.915
Scdf_P_ variance	0.578	0.523	0.795	0.608	0.279	0.267

Scdf_graphite_ mean	0.596	0.59	0.606	0.604	0.483	0.493
Scdf_graphite_ variance	0.06	0.058	0.064	0.063	0.02	0.023

Rort mean	1.386	1.403	1.405	1.401	1.298	1.284
Tort variance	0.004	0.005	0.007	0.008	0.003	0.002
